# The role of GABA in the regulation of GnRH neurons

**DOI:** 10.3389/fnins.2014.00387

**Published:** 2014-11-28

**Authors:** Miho Watanabe, Atsuo Fukuda, Junichi Nabekura

**Affiliations:** ^1^Department of Neurophysiology, Hamamatsu University School of MedicineHamamatsu, Japan; ^2^Department of Developmental Physiology, National Institute for Physiological SciencesOkazaki, Japan; ^3^Core Research for Evolutionary Science and Technology, Japan Science and Technology CorporationSaitama, Japan; ^4^Department of Physiological Sciences, The Graduate School for Advanced StudyHayama, Japan

**Keywords:** GnRH neuron, GABA, KCC2, NKCC1, LH surge

## Abstract

Gonadotropin-releasing hormone (GnRH) neurons form the final common pathway for the central regulation of reproduction. Gamma-amino butyric acid (GABA) has long been implicated as one of the major players in the regulation of GnRH neurons. Although GABA is typically an inhibitory neurotransmitter in the mature adult central nervous system, most mature GnRH neurons show the unusual characteristic of being excited by GABA. While many reports have provided much insight into the contribution of GABA to the activity of GnRH neurons, the precise physiological role of the excitatory action of GABA on GnRH neurons remains elusive. This brief review presents the current knowledge of the role of GABA signaling in GnRH neuronal activity. We also discuss the modulation of GABA signaling by neurotransmitters and neuromodulators and the functional consequence of GABAergic inputs to GnRH neurons in both the physiology and pathology of reproduction.

## Introduction

Gonadotropin-releasing hormone (GnRH) neurons constitute the final output neurons in the neuroendocrine control of reproduction (Freeman, 2006). Pulsatile GnRH release stimulates the secretion of the gonadotropins, luteinizing hormone (LH) and follicle-stimulating hormone (FSH) from the pituitary. LH and FSH stimulate the development of mature eggs and sperm and also the synthesis of the gonadal hormones; estrogen and progesterone from the ovaries and androgens from the testes. The gonadal steroids feedback to the hypothalamus and pituitary to decrease GnRH and gonadotropin secretion throughout the estrous cycle, except during the afternoon of proestrus, when elevated levels of estradiol, released by maturing ovarian follicles, initiate the preovulatory GnRH/LH surge that causes ovulation.

The hypothalamus contains a relatively small number of GnRH neurons and these are diffusely scattered throughout the hypothalamus. Hence the mechanisms enabling GnRH neurons to generate the discrete episodes of GnRH secretion remain unknown. GnRH release is closely related to the activity of GnRH neurons, which are regulated by neurotransmitters, steroid hormones, and growth factors (Freeman, 2006). GnRH neurons express both GABA_A_(Sim et al., 2000; Temple and Wray, 2005) and GABA_B_ receptors (Zhang et al., 2009) and receive GABAergic inputs that express estrogen receptors (Leranth et al., 1985); therefore, GABA has long been implicated as a major player in the regulation of GnRH neuron activity and secretion. In this brief review, we focus on the action of GABA on GnRH neurons.

## Excitatory and inhibitory actions of GABA

**Figure 1:**
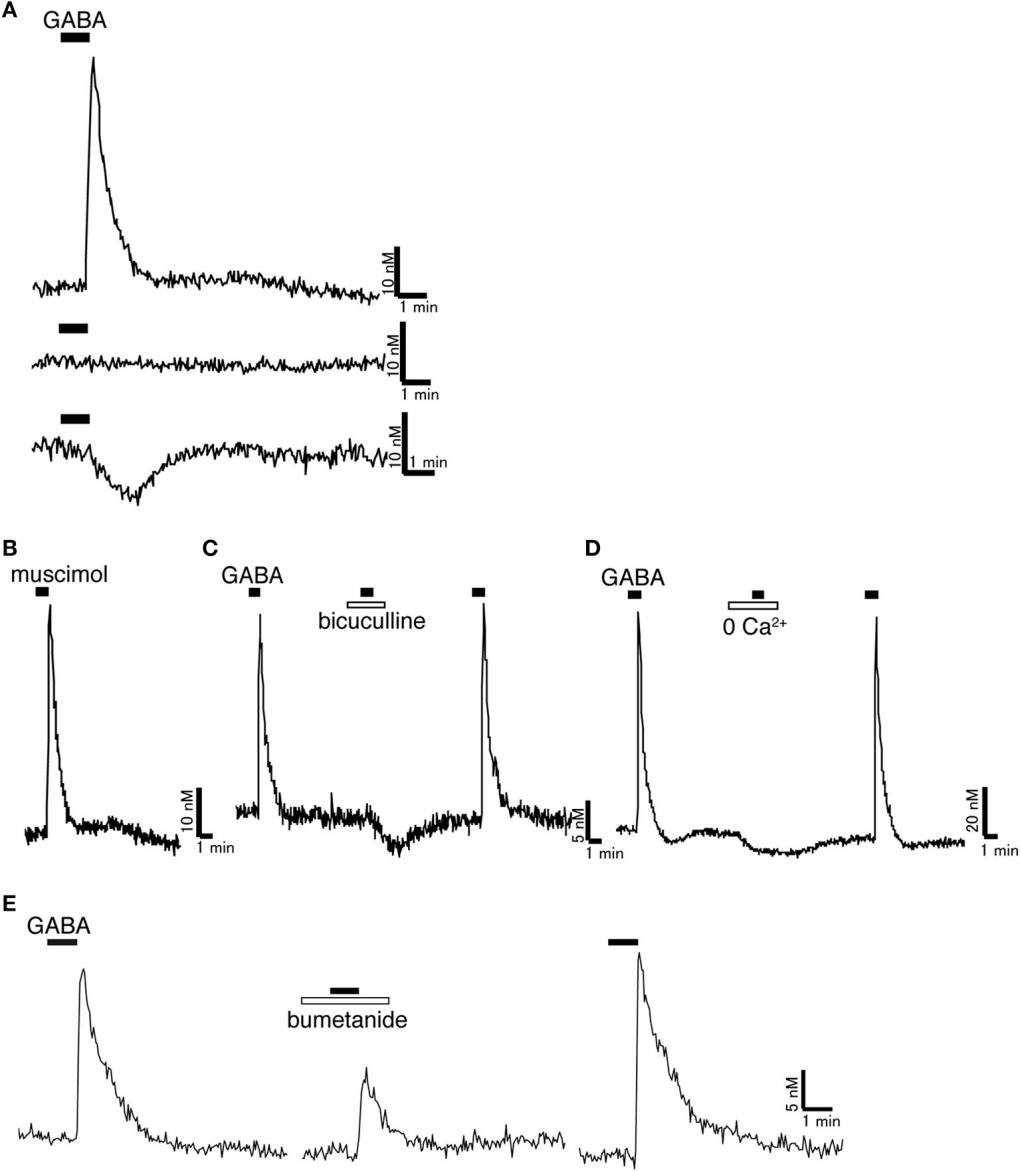
**Excitatory action of GABA on rat GnRH neurons. (A)** Representative [Ca^2+^]_i_ response to 100 μM GABA. Most GnRH neurons showed [Ca^2+^]_i_ increase in response to GABA. Some GnRH neurons did not respond to GABA. Some GnRH neurons showed [Ca^2+^]_i_ decrease in response to GABA. **(B)** Muscimol (100 μM), a GABA_A_ receptor agonist, increased [Ca^2+^]_i_ in GnRH neurons. **(C)** Bicuculline (100 μM), a GABA_A_ receptor antagonist, inhibited the [Ca^2+^]_i_ increase induced by GABA. **(D)** GABA-induced [Ca^2+^]_i_ increase was inhibited in Ca^2+^-free solution. **(E)** Bumetanide (100 μM), a blocker of NKCC1, reduced the GABA-induced [Ca^2+^]_i_ increase. Muscimol and GABA were applied as indicated with horizontal bars. Bicuculline, Ca^2+^-free solution, and bumetanide were applied as indicated with open bars (Originally published in Watanabe et al., 2009a).

The majority of *in vivo* whole animal studies have reported inhibitory actions of GABA on GnRH/LH secretion, although some reports have suggested excitatory effects of GABA (Donoso et al., 1992; Bilger et al., 2001). GABA infusion into the preoptic area or intraperitoneal injection of the GABA_A_ receptor agonist, muscimol, blocked the LH surge (Adler and Crowley, 1986; Herbison and Dyer, 1991), while GABA_A_ receptor antagonist, bicuculline, advanced the timing of the LH surge (Kimura and Jinnai, 1994). GABA release in the preoptic area was decreased prior to and during the time of the LH surge (Jarry et al., 1995). GABA is synthesized primarily from glutamate by the enzyme glutamate decarboxylase, two isoforms of which exist, GAD65 and GAD67 (Soghomonian and Martin, 1998). GAD67 mRNA levels in the preoptic area were decreased prior to the LH surge (Herbison et al., 1992). The number of terminals containing vesicular GABA transporter (VGAT, a marker of GABAergic neurons) was decreased in GnRH neurons at the time of the LH surge (Ottem et al., 2004). Injection of GABA or muscimol inhibited pulsatile LH release (Herbison et al., 1991; Jarry et al., 1991; Hiruma et al., 1994). The suppression of pulsatile LH release induced by infection stress was inhibited by bicuculline (Lin et al., 2012). From these *in vivo* studies, it is thought that GABA acts to inhibit the LH surge and pulsatile LH release via GABA_A_receptors. The origins of GABAergic inputs to GnRH neurons are not well established, but the anteroventral periventricular area (AVPV) (Ottem et al., 2004) and the suprachiasmatic nucleus (SCN) are candidate regions (Christian and Moenter, 2007). This is because GABAergic neurons both in the AVPV and SCN express ERα, while GABAergic neurons in the AVPV exhibit changes in GAD67 gene expression that parallel GABA release on the day of LH surge release (Curran-Rauhut and Petersen, 2002). However, from these experiments, it is difficult to clarify the direct actions of GABA on GnRH neurons. Because GnRH neurons lack any specific identifying morphology, and owing to their diffuse location (Herbison, 2006), it is difficult to directly study the cellular and molecular mechanisms in single, functional GnRH neurons. The direct action of GABA on GnRH neurons has been studied using an immortalized GnRH neuronal cell line (GT1). GT1 cells were generated by genetically targeted tumorigenesis in transgenic mice (Mellon et al., 1990). GT1 cells are thought to preserve many characteristics of native GnRH neurons. They generate spontaneous action potentials, exhibit transient oscillations of the intracellular Ca^2+^ concentration ([Ca^2+^]_i_) (Hales et al., 1994; Charles and Hales, 1995), and secrete GnRH in a pulsatile manner (Wetsel et al., 1992; Martínez de la Escalera et al., 1994). GT1 cells synthesize GABA (Ahnert-Hilger et al., 1998) and express functional GABA_A_ receptors (Favit et al., 1993). The activation of GABA_A_ receptors in GT1 cells depolarizes the membrane potential, which activates voltage-gated Ca^2+^ channels, thereby facilitating Ca^2+^ influx and increasing [Ca^2+^]_i_ and GnRH release (Favit et al., 1993; Hales et al., 1994; Martínez de la Escalera et al., 1994; Spergel et al., 1995). Although GT1 cells are still useful, especially in biochemical and molecular biology experiments, which often require many cells with uniform characteristics, the immortalized nature of these cells may interfere with normal differentiated functions and the study of the neural circuitry that regulates GnRH neurons, such as afferent inputs, cannot be accomplished in GT1 cells. To overcome these barriers, transgenic mice and rats expressing enhanced green fluorescent protein (EGFP) under the control of the GnRH promoter were generated (Spergel et al., 1999; Suter et al., 2000; Watanabe et al., 2009a). Using these mice and rats, the direct action of GABA on EGFP-tagged living GnRH neurons has been studied (Herbison and Moenter, 2011). Activation of GABA_A_ receptors excited mouse GnRH neurons in acutely prepared slices through the hypothalamus (DeFazio et al., 2002) and evoked increases in [Ca^2+^]_i_ in GnRH-Pericam transgenic mice (Constantin et al., 2010). The reversal potential of GABA_A_ receptor current (E_GABA_) was more positive than the resting potential in mouse GnRH neurons (E_GABA_ = −36.5 ± 1.2 mV, V_rest_ = −50.7 ± 1.7 mV) (DeFazio et al., 2002). Therefore, GABA caused depolarization in GnRH neurons. The GABA_A_ receptor antagonist, bicuculline, or picrotoxin decreased the firing rate of GnRH neurons in the presence of ionotropic glutamate receptor antagonists, AP5 and CNQX, which excluded glutamatergic transmission (Moenter and DeFazio, 2005). Activation of somatic/proximal dendritic GABA_A_ receptors in GnRH neurons caused robust action potential discharges by the activation of L-type calcium channels (Hemond et al., 2012). Furthermore, activation of GABA_A_ receptors increased [Ca^2+^]_i_ in isolated GnRH neurons from prepubertal and adult rats (Watanabe et al., 2009a) (Figure 1). Bicuculline inhibited the [Ca^2+^]_i_ increase induced by GABA. GABA-induced [Ca^2+^]_i_ increase was inhibited in Ca^2+^-free solution. E_GABA_ of rat adult GnRH neurons was more positive than resting potential (E_GABA_ = −26 ± 1.4 mV, V_rest_ = −60 to −50 mV) (Yin et al., 2008). Therefore, GABA also depolarized rat GnRH neurons. However, contradictory results on the actions of GABA have been demonstrated using transgenic mice in which GnRH neurons express beta-galactosidase (GnRH-lacZ mice). The beta-galactosidase can convert substrates to a fluorescent state enabling the visualization of GnRH neurons. The effect of GABA on GnRH neurons switched from depolarization to hyperpolarization at puberty in females (Han et al., 2002). A GABA_A_ receptor antagonist increased the firing rate of GnRH neurons (Han et al., 2004); however, the recording was performed in the absence of CNQX and AP5. The GABA_A_ receptor antagonist acts on all cells in the brain slice; therefore, it removes GABAergic inhibitory signaling and causes disinhibition in most neurons. Therefore, to remove the effect of disinhibition of glutamatergic neurons in the network that regulates GnRH neurons, glutamatergic signaling needs to be blocked. The presence of a tonic GABA_A_ receptor current in GnRH neurons was also reported as inhibitory. GABA and THIP, a GABA_A_ δ receptor agonist, hyperpolarized the membrane potential in adult GnRH neurons (Bhattarai et al., 2011). GABA has also been reported to act to GnRH neurons at the level of GnRH nerve terminals in the median eminence. The conditional activation of GABA release near GnRH nerve terminals disrupted the estrous cycle and reduced fertility in rats (Bilger et al., 2001). Recent reports show that GnRH neurons have unique morphology; long-distance projections to the median eminence function simultaneously as axons and dendrites (Herde et al., 2013). These GnRH projections have functional GABA_A_ receptors and the activation of GABA_A_ receptors depolarized the membrane potential and initiated action potentials at the median eminence. GABA is also excitatory to GnRH neurons in a variety of species, such as goldfish and sea lamprey (Trudeau et al., 2000; Reed et al., 2002; Root et al., 2004; Popesku et al., 2008). In an adult teleost fish, the dwarf gourami, GABA_A_ receptor activation induced excitation in the terminal nerve-GnRH neurons (Nakane and Oka, 2010). From these results, GABA might regulate the excitability of GnRH neurons at GnRH cell bodies as well as at the median eminence.

Recently, the first electrical recording of GnRH neurons *in vivo* in the anesthetized mouse was reported. Whereas muscimol evoked excitatory, inhibitory, or mixed effects on GnRH neuron firing, picrotoxin resulted in a consistent suppression of firing (Constantin et al., 2013). This study also reported that the effects of GABA on GnRH neurons were critically dependent upon the orientation within the slice (Constantin et al., 2012b). GABA was excitatory to GnRH neurons in coronal slices but inhibitory in the anterior hypothalamic area in horizontal slices. This is because of the direct activation of GABA_A_ or GABA_B_ receptors. GABA_B_ receptors also modulate the excitability of GnRH neurons. GABA_B_ R1 and R2 subunits are expressed in GnRH neurons (Zhang et al., 2009), and the GABA_B_ receptor agonist baclofen hyperpolarized GnRH neurons through activation of an inwardly rectifying K^+^ current (Lagrange et al.^1995^; Zhang et al., 2009). Therefore, the net GABA effects are likely to be determined by the balance of GABA_A_ vs. GABA_B_ receptor-mediated effects along the GnRH neuron soma and dendrite (Constantin et al., 2013).

Few studies have investigated the effect of GABA on gene expression in GnRH neurons. Intracerebroventricular injection of muscimol induced a pronounced decrease of GnRH mRNA levels in the preoptic area. Injection of baclofen had no effect on GnRH mRNA levels (Bergen et al., 1991; Leonhardt et al., 1999). But opposite results have also been reported (Kang et al., 1995; Cho and Kim, 1997). Further work is needed to clarify this point.

From these results, although the action of GABA on GnRH neurons is still controversial, most GnRH neurons appear to be excited by GABA. However, GnRH neurons may exhibit heterogeneity in their GABA response depending on their location in the hypothalamus. Clarification of this point requires further study.

## [Cl^−^]_i_ determines the polarity of the GABA response

**Figure 2:**
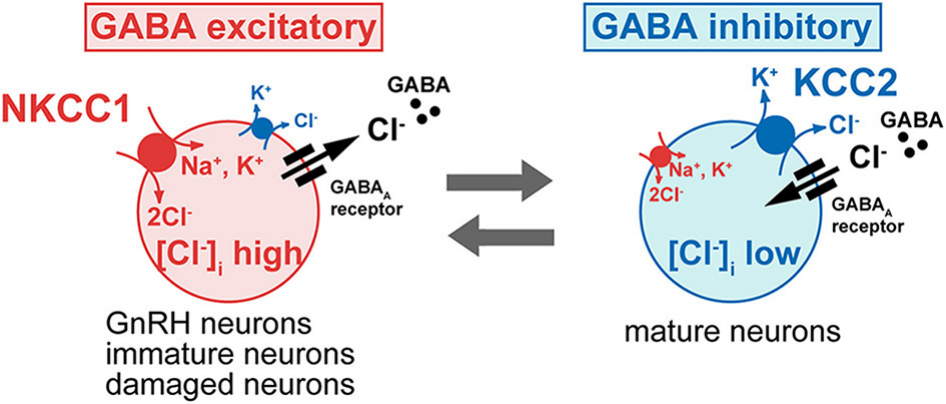
**The intracellular Cl^−^ concentration determines the polarity of GABA response**. The GABA action is excitatory in immature neurons because [Cl^−^]_i_ is high, owing to high levels of the Na^+^-K^+^-2Cl^−^ cotransporter (NKCC1), which mediates inward transport of Cl^−^, and to low levels of the K^+^-Cl^−^ cotransporter (KCC2), which excludes Cl^−^ from the cell. In most neurons, the GABA response switches from excitation to inhibition during early postnatal development, due to the developmental decrease of the NKCC1 and increase of the KCC2. However, even in the mature neurons, neuronal damage down regulates the KCC2 and elevated Cl^−^ concentration shifts GABA response from hyperpolarization to depolarization, occasionally excitation. Most GnRH neurons show the unusual characteristic of being excited by GABA in the adult brain.

Because Cl^−^ is the most permeant ion through the GABA_A_ receptor ion channel, the intracellular Cl^−^ concentration ([Cl^−^]_i_) determines the polarity of the GABA response (Ben-Ari, 2002). A hyperpolarizing and generally inhibitory action of GABA occurs when [Cl^−^]_i_ is low, whereas a depolarizing and generally excitatory action of GABA is seen when [Cl^−^]_i_ is high. In most neurons, the GABA response switches from a depolarization to a hyperpolarization during early postnatal development. Among the many molecules involved in [Cl^−^]_i_ homeostasis, the exclusively neuronal subtype of the K^+^-Cl^−^ cotransporter (KCC2), which couples the K^+^ electrochemical gradient to Cl^−^ extrusion, is the principal molecule which maintains low [Cl^−^]_i_ in mature neurons (Blaesse et al., 2009). In contrast, the neuronal subtype of the Na^+^-K^+^-2Cl^−^ cotransporter (NKCC1), which mediates inward transport of Cl^−^, maintains high [Cl^−^]_i_ (Figure 2). Because GABA excites in most GnRH neurons, one would predict the expression of KCC2 to be low and that of NKCC1 to be high in GnRH neurons. Actually, bumetanide, a blocker of NKCC1, reduced the GABA-induced [Ca^2+^]_i_ increase in rat GnRH neurons (Figure 1). GT1 cells do not express detectable levels of KCC2 mRNA or protein but do express NKCC1 mRNA and protein (DeFazio et al., 2002). Adult rat GnRH neurons do not express KCC2 protein and express low levels of NKCC1 protein. KCC2 mRNA was expressed in 4.9% of GnRH neurons, whereas NKCC1 mRNA was expressed in 13.5% of GnRH neurons (DeFazio et al., 2002). A similar expression of KCC2 and NKCC1 mRNAs was shown in adult mouse GnRH neurons. The vast majority of KCC2 and NKCC1 expressing GnRH neurons are located in the anterior region of the preoptic area where the greatest concentration of neuroendocrine GnRH neurons is normally observed. Heterogeneous expression of KCC2 in mouse GnRH neurons has also been reported with 34% of GnRH neurons expressing KCC2 mRNA (Leupen et al., 2003). This proportion was similar in females and males. However, females exhibited a marked rostrocaudal gradient of colocalization that was not seen in males. Protein levels and the function of KCC2 and NKCC1 are rapidly modulated by intracellular and extracellular substrates (Blaesse et al., 2009). The activity, cell surface stability, and membrane trafficking of KCC2 are modulated by the phosphorylation of serine, threonine, and tyrosine residues in the C terminal region (Watanabe et al., 2009b; Kahle et al., 2013). KCC2 expression levels are reduced in response to various pathophysiological conditions (Kahle et al., 2008), including axotomy (Nabekura et al., 2002; Toyoda et al., 2003), global ischemia (Reid et al., 2000) chronic pain (Eto et al., 2012), interictal activity (Rivera et al., 2004), and neuronal stress (Wake et al., 2007) with resulting increases in [Cl^−^]_i_ and a shift of GABA-mediated responses from hyperpolarizing to depolarizing. Therefore, it is reasonable to speculate that the functional expression of NKCC1 and/or KCC2 is changed according to estrous cycle stage and is different between males and females. These changes may modulate the response to GABA in GnRH neurons. Further studies are needed to clarify this point. In immature or injured neurons when GABA is also excitatory, this excitation can result in action potentials, [Ca^2+^]_i_ oscillations, and synchronized patterns of activity (Ben-Ari, 2002; Toyoda et al., 2003). GnRH neurons also show spontaneous activity and [Ca^2+^]_i_ oscillations (Constantin et al., 2012a) and the frequency of calcium oscillation in GnRH neurons was reduced by a GABA_A_ receptor antagonist. Therefore, the excitatory action of GABA in GnRH neurons may contribute to the synchronous activity that generates discrete episodes of GnRH secretion.

## Modulation of GABA transmission

Several neurotransmitters have been reported to regulate the activity of GABA neurons. Kisspeptin is a potent stimulator of GnRH release via G protein-coupled receptor 54 (GPR54) (Gottsch et al., 2004; Dungan et al., 2007; Mayer and Boehm, 2011). GnRH neurons express GPR54 (Messager et al., 2005) and kisspeptin acts directly on GnRH neurons (Han et al., 2005; Pielecka-Fortuna and Moenter, 2010). Kisspeptin also acts indirectly to modulate GnRH neurons via a change in GABAergic transmission. Kisspeptin increased the frequency and amplitude of GABAergic postsynaptic currents in GnRH neurons in an estradiol-dependent manner at the time of estradiol negative feedback (Pielecka-Fortuna and Moenter, 2010). Metabotropic glutamate receptors (mGluRs) also regulate GABA transmission to GnRH neurons. The endogenous activation of presynaptic mGluRs decreased the frequency of GABA_A_-mediated spontaneous postsynaptic currents in GnRH neurons and decreased GnRH neuron firing rate (Chu and Moenter, 2005). These effects occur through group II/III mGluRs and are mimicked by GnRH neural activity, suggesting a role for mGluRs in feedback regulation. The adipose-derived hormone, leptin, regulates GABAergic signaling. Acute fasting decreased the frequency of spontaneous GABAergic postsynaptic currents in GnRH neurons and GnRH neuronal activity (Sullivan et al., 2003; Sullivan and Moenter, 2004a). Because GnRH neurons do not express leptin receptors, the leptin effect was indirect (Quennell et al., 2009). GABAergic signaling seems to communicate information about metabolic status to the GnRH neurons indirectly. Retrograde endocannabinoid signaling reduces GABAergic synaptic transmission to GnRH neurons via the activation of presynaptic CB1 receptors, resulting in inhibition of GnRH neuron firing activity (Farkas et al., 2010). The depolarization of GnRH neurons induced short-term inhibition of GABAergic afferents via endocannabinoids and glia derived prostaglandins, and this interaction was steroid and likely sex dependent (Glanowska and Moenter, 2011). GnRH itself also regulated the activity of GABA neurons. GABAergic neurons express the type-1 GnRH receptor. Low levels of GnRH reduced the frequency of GABAergic postsynaptic currents in GnRH neurons, suggesting that low-dose GnRH suppressed GnRH firing in part by decreasing GABAergic transmission to GnRH neurons (Chen and Moenter, 2009). The pineal hormone, melatonin, is involved in the regulation of reproductive function, including the timing of the LH surge. Melatonin modulates GABA_A_ receptor currents in GnRH neurons isolated from GnRH-EGFP transgenic rats, positively in males and negatively in females (Sato et al., 2008).

GABAergic transmission is also regulated by a nonclassical action of the ovarian steroid, estradiol. Estrogen receptor α (ERα) agonists reduced the frequency of GABA transmission to GnRH neurons (Chu et al., 2009). A nonclassical action of estradiol via ERα on GnRH neurons that caused phosphorylation of ERK1/2 and consequently CREB was blocked by a GABA_A_ receptor antagonist (Kwakowsky et al., 2014). In contrast, ERβ agonists increased GABA transmission and postsynaptic response. Estrogen interacted with the classical ERα at the level of the GABAergic nerve terminal to regulate action potential-independent GABA release (Romanò et al., 2008). Steroid metabolites known as neurosteroids also modulate the function of the GABA_A_ receptor. Specifically, the progesterone derivative allopregnanolone is an allosteric agonist, whereas the androgen, dehydroepiandrosterone sulfate (DHEAS), is an allosteric antagonist. Allopregnanolone increased GABAergic miniature postsynaptic current frequency, amplitude and decay time. DHEAS reduced mPSC frequency and amplitude but did not alter decay time (Sullivan and Moenter, 2003). Also, in rat GnRH neurons, GABA_A_ currents were augmented by allopregnanolone and 3α,21-dihydroxy-5α-pregnan-20-one (Yin et al., 2008).

Therefore, several neurotransmitters and hormones modulate GABAergic transmission to GnRH neurons, and this modulation may mediate various physiological stimuli that regulate GnRH neuronal activity.

## Functional role of GABA action on GnRH neurons

The precise physiological role of direct GABA_A_ receptor activation in GnRH neurons *in vivo* remains to be investigated. Although the near complete abolition of GABA_A_ receptor signaling by knockout of the GABA_A_ receptor γ2 subunit in GnRH neurons was found to have no major effect on fertility *in vivo* (Lee et al., 2010), there are many reports that propose a role for GABA in multiple aspects of GnRH neuronal physiology. These range from embryonic migration to a role in puberty and both estrogen negative and positive feedback.

GABA plays a key developmental role in the regulation of GnRH neuron migration from the olfactory placodes into the forebrain during fetal development. Like GT1 cells, a subset of embryonic GnRH neurons can produce GABA during migration (Tobet et al., 1996; Ahnert-Hilger et al., 1998). GABA is also present in cells and fibers along the GnRH migratory route throughout the nasal compartment (Tobet et al., 1996; Wray et al., 1996). GAD65 is expressed exclusively in undifferentiated neuronal progenitors confined to the proliferative zones of the sensory vomeronasal and olfactory epithelia (Vastagh et al., 2014). In contrast, GAD67 is expressed in a subregion of the nonsensory epithelium/vomeronasal organ epithelium containing the putative GnRH progenitors and GnRH neurons migrating from this region. Muscimol inhibited GnRH neuron migration and decreased extension of GnRH fibers. Bicuculline led to a disorganized distribution of GnRH neurons in the forebrain (Bless et al., 2000). Transgenic mice that selectively over-express GAD67 in GnRH neurons had more GnRH neurons in aberrant locations in the cerebral cortex and fewer neurons reaching the forebrain (Heger et al., 2003). Consequently, hypothalamic GnRH content was low during the second postnatal week, while in adult mice disrupted the estrous cycle and litter sizes were reduced. In contrast, in GABA deficient mice (GAD 67 knockout mice), GnRH neurons reached the nasal/forebrain junction earlier and entered the forebrain earlier (Lee et al., 2008). From these results, GABA production within GnRH neurons alters the migratory fate of these neurons and the timely termination of GABA production within the GnRH neuronal network is required for normal reproductive function. The role of GABAergic inputs on GnRH neuronal migration was also evaluated using olfactory explants. Mouse embryonic GnRH neurons in olfactory pit explant cultures express GABA_A_ receptors and activation of GABA_A_ receptors resulted in membrane depolarization (Kusano et al., 1995) and increased [Ca^2+^]_i_ (Moore and Wray, 2000). Muscimol inhibited GnRH migration and bicuculline or picrotoxin increased GnRH migration (Fueshko et al., 1998). Stromal derived growth factor (SDF-1) and GABA synergistically regulate the rate of GnRH migration (Casoni et al., 2012). SDF-1 accelerated migration by hyperpolarization via changes in potassium, while GABA slowed migration by depolarization via changes in chloride. These studies demonstrate that GABAergic activity in nasal regions has effects on migration of GnRH neurons and that GABA participates in appropriate timing of GnRH neuronal migration into the developing brain.

GABA has been reported to have a role in mediating puberty. In most neurons of the central nervous system, the GABA response switches from a depolarization to a hyperpolarization during early postnatal development (Ben-Ari, 2002). One group reported that the switch of GABA_A_ receptor signaling in GnRH neurons was delayed until the time of puberty (Han et al., 2002). The expression patterns of GABA_A_ receptor subunit mRNAs in GnRH neurons change during the developmental period. In juvenile and prepubertal female mice, α1-5, β 1-3, and γ2,3 subunits are broadly expressed in a heterogeneous manner. Adult female mouse GnRH neurons of the rostral preoptic area express predominantly α1, α5, β 1, and γ2 subunits and those of the medial septum express α1, α3, α5, β 1, β 3, and γ2 subunits (Sim et al., 2000). These changes appear to involve the activation of the GnRH neurons at puberty. In female rhesus monkeys, a reduction of GABA inhibition is thought to be critical for the mechanism initiating puberty onset, because chronic infusion of bicuculline into the stalk-median eminence significantly increased GnRH release and accelerated the timing of the menarche and first ovulation (Terasawa et al., 2011). Bicuculline dramatically stimulated kisspeptin release in the medial basal hypothalamus of prepubertal monkeys but had little effect on kisspeptin release in midpubertal monkeys (Kurian et al., 2012). This implies that a reduction in tonic GABA inhibition of GnRH release is, at least in part, mediated through kisspeptin neurons.

GABA plays a critical role in mediating both estradiol negative and positive feedback and appears to control the timing of the switch in estradiol feedback action. The frequency of GABA transmission to GnRH neurons is directly correlated with estradiol negative and positive feedback. Frequency of GABAergic postsynaptic currents was low during negative feedback but frequency and amplitude of GABAergic postsynaptic currents was increased at surge onset (Christian and Moenter, 2007). This indicates that estradiol induces diurnal shifts in GABA transmission at appropriate times to generate changes in GnRH neuronal firing activity and hormone release characteristic of both negative and positive feedback. Adult mice lacking functional GABA_B_ receptors (GABA_B1_KO) displayed disruption of cyclicity and fertility (Catalano et al., 2005). GABA_B1_KO mice showed increased *Gnrh1* and *Gad1* expression but decreased *Kiss1* expression in the medial basal hypothalamus of neonatal mice (Di Giorgio et al., 2013). Thus, GABA signaling via GABA_B_ receptors is also important for regulating the estrous cycle.

Metabolic signals have influences on fertility. GABA neuron-specific leptin receptor knock-out female and male mice show significantly delayed puberty onset (Zuure et al., 2013). Female mice lacking functional leptin receptors in GABAergic neurons have hypogonadotropic hypogonadism (Martin et al., 2014). Adult leptin receptor knockout mice showed decreased fecundity. There results suggest that leptin signaling in GABAergic neurons plays a critical role in the timing of puberty onset and is involved in fertility regulation. Therefore, GABAergic afferents integrate metabolic signals for delivery to GnRH neurons.

In human, GABAergic axons exhibiting VGAT immunoreactivity innervate the soma and dendrites of GnRH neurons (Hrabovszky et al., 2012). A change in GABAergic transmission is associated with the hypothalamic abnormalities of fertility disorders. In polycystic ovary syndrome model mice, which were exposed to androgen *in utero*, the size and frequency of GABAergic postsynaptic currents were increased (Sullivan and Moenter, 2004b). From these data, increased GnRH pulse frequency observed in polycystic ovary syndrome may be attributable to androgen-induced increases in GABAergic drive to GnRH neurons.

Although the importance of GABAergic inputs has been demonstrated in *in vitro* studies, further work is needed to determine the precise functional roles of direct GABAergic inputs to GnRH neurons *in vivo*. Because most GnRH neurons show the unusual characteristic of being excited by GABA, the excitatory action of GABA might make a major contribution to the regulation of GnRH neuron activity and secretion. As aberrant central GABAergic signaling is seen in polycystic ovary syndrome model mice, change in neuronal GABA activity appears to alter reproductive status both physiologically and pathologically. Therefore, determination of the precise role of GABAergic transmission in the regulation of GnRH neurons is important for understanding the regulation of normal reproduction as well as the hypothalamic abnormalities of fertility disorders.

### Conflict of interest statement

The authors declare that the research was conducted in the absence of any commercial or financial relationships that could be construed as a potential conflict of interest.
